# Degradation of Postsynaptic Scaffold GKAP and Regulation of Dendritic Spine Morphology by the TRIM3 Ubiquitin Ligase in Rat Hippocampal Neurons

**DOI:** 10.1371/journal.pone.0009842

**Published:** 2010-03-24

**Authors:** Albert Y. Hung, Clifford C. Sung, Ilana L. Brito, Morgan Sheng

**Affiliations:** 1 Departments of Brain and Cognitive Sciences and Biology, The Picower Institute for Learning and Memory, Massachusetts Institute of Technology, Cambridge, Massachusetts, United States of America; 2 Department of Neurology, Massachusetts General Hospital, Boston, Massachusetts, United States of America; The Research Center of Neurobiology-Neurophysiology of Marseille, France

## Abstract

Changes in neuronal activity modify the structure of dendritic spines and alter the function and protein composition of synapses. Regulated degradation of postsynaptic density (PSD) proteins by the ubiquitin-proteasome system is believed to play an important role in activity-dependent synaptic remodeling. Stimulating neuronal activity in vitro and in vivo induces the ubiquitination and degradation of GKAP/SAPAP and Shank, major scaffold proteins of the PSD. However, the specific ubiquitin ligases that regulate postsynaptic protein composition have not been identified. Here we identify the RING finger-containing protein TRIM3 as a specific E3 ubiquitin ligase for the PSD scaffold GKAP/SAPAP1. Present in PSD fractions from rat brain, TRIM3 stimulates ubiquitination and proteasome-dependent degradation of GKAP, and induces the loss of GKAP and associated scaffold Shank1 from postsynaptic sites. Suppression of endogenous TRIM3 by RNA interference (RNAi) results in increased accumulation of GKAP and Shank1 at synapses, as well as enlargement of dendritic spine heads. RNAi of TRIM3 also prevented the loss of GKAP induced by synaptic activity. Thus, TRIM3 is a novel E3 ligase that mediates activity-dependent turnover of PSD scaffold proteins and is a negative regulator of dendritic spine morphology.

## Introduction

In mammalian neurons, excitatory synaptic transmission occurs primarily at specialized, actin-rich protrusions called dendritic spines. Localized to the postsynaptic membrane of spines is the postsynaptic density (PSD), a complex protein apparatus comprised of glutamate receptors, associated scaffold and cytoskeletal proteins, and signal transduction molecules. Synaptic activity induces growth and structural rearrangements of the PSD and dynamic turnover of PSD proteins [1], [2]. Chronic alterations in synaptic activity result in a coordinated, reversible change in postsynaptic protein composition, with increased activity stimulating degradation of a subset of PSD proteins [3]. Dendritic spines undergo bidirectional morphological changes in response to neuronal activity, and the morphology of individual spines is correlated with synaptic strength and their capacity to undergo activity-dependent plasticity [4]–[6]. Thus, regulated turnover of PSD proteins may be one mechanism for determining synaptic strength and plasticity.

The ubiquitin-proteasome system (UPS) has emerged as an important mediator of synaptic protein degradation [7], [8]. Proteins are generally targeted for degradation by the UPS by the covalent addition of polyubiquitin chains. Ubiquitination requires a series of enzymatic reactions, catalyzed sequentially by E1, E2, and E3 enzymes. E3 ubiquitin ligases, hundreds of which exist in mammalian genomes, appear to recognize specific proteins and thus determine the specificity of protein substrates targeted for UPS-mediated degradation [9]. While the abundance of many proteins in the PSD is regulated by UPS activity, only a small number of postsynaptic proteins have been observed to undergo activity-dependent ubiquitination, including the two abundant scaffold protein families called Shank and GKAP/SAPAP [3]. By targeting specific substrates that serve as the structural core of the PSD, the UPS may regulate a larger set of postsynaptic proteins.

The TRIM/RBCC proteins are a class of putative single subunit E3 ubiquitin ligases characterized by the presence of a tripartite motif containing a RING finger, one or two zinc-binding B-box domains, and an associated coiled-coil domain [10], [11]. This class of proteins, encoded by at least 68 genes in the human genome, has been implicated in a variety of cellular processes and may play a role in some human diseases. Substrates for the ubiquitin ligase activity of a number of TRIM proteins have been identified [10]; for example, TRIM18/Mid2, implicated as the cause of X-linked Opitz syndrome, degrades protein phosphatase 2A [12]. The TRIM family member TRIM3 was originally identified as a brain-enriched RING finger protein (BERP) [13]. It is highly expressed in brain, and is reported to interact with myosin Vb and the actin-binding protein α-actinin-4 [13], [14]. However, the E3 ligase activity of TRIM3 has not been established and its substrates are unknown.

The GKAP (also known as SAPAP) and Shank family of proteins are abundant interacting scaffolds of the PSD [2], [15]. By binding to both Shank and PSD-95, GKAP links Shank to the PSD-95 protein complex, including NMDA-type glutamate receptors [16], [17] GKAP and Shank protein levels in synapses are regulated by activity and they are among the most prominently ubiquitinated proteins in the PSD [3]. In this study, we identify TRIM3 as a specific E3 ligase for GKAP. TRIM3 induces ubiquitination and proteasome-dependent degradation of GKAP in heterologous cells. Overexpression of TRIM3 in hippocampal neurons suppresses GKAP protein levels, concomitant with loss of Shank1 and reduced spine head width. Conversely, RNAi knockdown of TRIM3 resulted in an accumulation of postsynaptic GKAP and Shank and spine head enlargement. We also present evidence that TRIM3 is involved in the activity-dependent turnover of GKAP. These results identify a novel ubiquitin ligase involved in regulation of PSD composition and dendritic spine morphology.

## Results

### Identification of TRIM3 as a putative postsynaptic regulator by RNAi screen

To identify specific E3 ligases that mediate degradation of PSD proteins, we performed an RNAi screen of a subset of known and putative E3 enzymes in primary hippocampal neurons. The initial screen targeted cullins 1, 2, 3, 4A and 5, core subunits of a superfamily of multisubunit ubiquitin ligases [18]; APC2, a cullin-related subunit of the anaphase-promoting complex [19]; E6-AP (also known as Ube3A), a single subunit E3 whose mutation underlies a human mental retardation syndrome called Angelman syndrome [20]; and Mdm2, an E3 that was reported to ubiquitinate PSD-95 [21]. In addition, we included two members of the TRIM/RBCC family of putative RING finger E3 ligases -- TRIM3 and TRIM9 -- that have been detected in the PSD by mass spectrometry [22]. Three different shRNAs were designed against each candidate E3 (except APC2, which had two shRNAs) and expressed from the plasmid vector pSUPER [23].

If a specific E3 mediates the ubiquitination and degradation of a PSD protein, then that PSD protein should accumulate to higher levels when expression of the endogenous E3 is suppressed by RNAi. For the initial screen, we transfected pools of RNAi constructs targeting individual E3 candidates together with a “reporter” PSD protein construct -- GFP-tagged Shank1A, a full-length splice variant of Shank1 [16], [24]. In addition to being itself a target of UPS degradation [3], Shank1A is localized to synapses, and its accumulation requires other scaffold proteins in the PSD, including GKAP, Homer, and PSD-95 [16], [17], [25]. Screening with the GFP-tagged reporter offers a technical advantage over endogenous Shank1 staining because the GFP fluorescence necessarily stems from the neuron transfected with the RNAi constructs and reduces experimental variability introduced by immunostaining. Thus, we reasoned that an increase of GFP-Shank1A at postsynaptic sites would reflect impaired degradation of Shank1A itself or of other proteins controlling the size of the PSD. Cultured rat hippocampal neurons were transfected at 14 days in vitro (DIV 14) with GFP-Shank1A and pools of RNAi constructs targeted against each individual E3. After three days, GFP-Shank1A fluorescence intensity was measured in transfected cells in “blinded” fashion.

**Figure 1:**
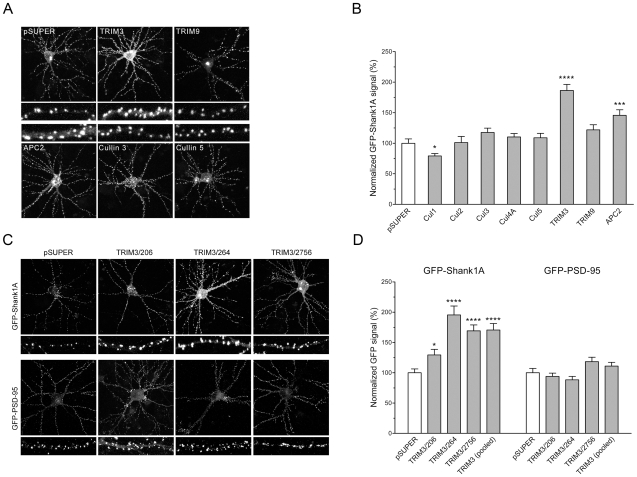
RNA interference-based screen for E3 ubiquitin ligases regulating GFP-Shank1A expression. (A) Primary rat hippocampal neurons (DIV 14) were cotransfected with GFP-tagged Shank1A and either empty vector (pSUPER) or pools of RNAi plasmids targeting various E3 ligases, as indicated. Images show GFP-Shank1A fluorescence. High magnification panels show representative dendritic segments. (B) Quantification of GFP-Shank1A fluorescence in transfected neurons, normalized to signal in pSUPER-transfected cells within the same experiment. Histograms (mean ± SEM) show average GFP intensity for cells from 3–4 independent experiments. (C) Effect of different individual TRIM3 RNAi constructs on expression of cotransfected GFP-Shank1A or GFP-PSD-95. (D) Quantification of GFP-Shank1A or GFP-PSD-95 signal from neurons cotransfected with individual TRIM3 RNAi plasmids, normalized to pSUPER vector control. p-values are calculated relative to pSUPER control. *p<0.05, ***p<0.001, ****p<0.0001, t-test.

In transfected neurons, GFP-Shank1A accumulated in a punctate spiny distribution along dendrites consistent with a postsynaptic localization (Figure 1A), and as previously reported [26]. The majority of pooled RNAi constructs had no effect on the pattern or fluorescence intensity levels of the GFP-Shank1A reporter. However, cotransfection with the pooled TRIM3 shRNA constructs led to a highly significant increase in GFP-Shank1A fluorescence (Figure 1A, B). APC2 knockdown also had a similar, albeit less robust, effect. With RNAi pools targeting E6-AP/Ube3A or Mdm2, only a small number of transfected neurons could be identified, all of which appeared unhealthy; thus, these were excluded from further analysis (data not shown).

To corroborate the TRIM3 result, we next tested the three TRIM3 shRNA plasmids separately. Neurons transfected with each of the individual TRIM3 shRNA plasmids showed increased GFP-Shank1A fluorescence; the biggest effect was seen with construct TRIM3/264. (Figure 1C, D). GFP-PSD-95 also targets to postsynaptic sites when transfected into hippocampal neurons (Figure 1C). However, in contrast to their effect on GFP-Shank1A, none of the TRIM3 RNAi plasmids affected the pattern or intensity of overexpressed GFP-PSD-95. The fact that TRIM3 knockdown shows some selectivity in its effect on postsynaptic scaffold proteins, and that it *increases* postsynaptic accumulation of GFP-Shank1A, would argue against a non-specific toxic effect of TRIM3 RNAi in neurons.

Using an antibody against the N-terminal domain of TRIM3 that does not cross-react with TRIM9 or the highly homologous TRIM2 protein (Figure S1A), we examined the effects of TRIM3 RNAi on the expression of TRIM3 in cultured hippocampal neurons. Each of the TRIM3 RNAi constructs reduced the immunostaining of endogenous TRIM3 in both cell body and dendrites of transfected cells (Figure S1B). The knockdown of TRIM3 expression by the TRIM3/2756 shRNA (which is directed against the 3′ untranslated region) could be rescued by overexpression of wildtype TRIM3 lacking its native 3′ UTR. RNAi constructs targeting TRIM2 or zinc transporter ZnT3 did not affect TRIM3 staining in neurons. As expected, the TRIM3/264 RNAi construct also decreased the expression of cotransfected TRIM3 protein in COS7 cells, while having no effect on the related protein TRIM2 (Figure S1C).

### Expression and distribution of TRIM3 in brain and cultured neurons

**Figure 2:**
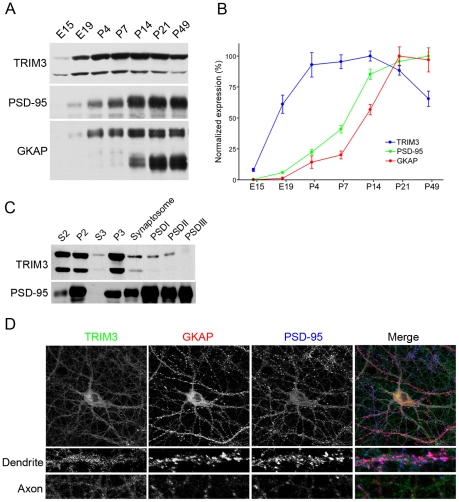
Expression and distribution of TRIM3 in cortex and cultured neurons. (A) Developmental expression of TRIM3. Total homogenates were prepared from rat forebrain at various ages. Equal amounts of protein were immunoblotted for TRIM3, PSD-95 or GKAP. (B) Immunoblots were quantified by densitometry in triplicate or quadruplicate. Signals were normalized to peak expression level. For TRIM3 and GKAP, the plotted signal represents the sum of multiple protein bands. Mean ± SEM is shown. (C) Biochemical fractionation of adult rat cerebral cortex. Protein amounts loaded were 30 µg/lane (TRIM3) or 10 µg/lane (PSD-95) for S2, P2, S3, P3, and synaptosomes, and 7.5 µg/lane (TRIM3) or 2.5 µg/lane (PSD-95) for PSDI, -II, -III samples. (D) Cultured rat hippocampal neurons (DIV 19) were immunostained for TRIM3 (green), GKAP (red) and PSD-95 (blue). High magnification panels show representative dendritic and axon segments.

What is the expression pattern of TRIM3 in neurons? Previous studies showed that TRIM3 mRNA expression is highest in the brain, moderate in the lung and low in liver, kidney, and heart [13]. By immunoblotting, TRIM3 was expressed in the brain at low levels at embryonic day 15 (E15) and then increased during the first two postnatal weeks before decreasing through adulthood (Figure 2A, B). The pattern differed from the steeply rising expression of PSD-95 and GKAP during postnatal development (Figure 2A, B). Two major bands were detected in TRIM3 Western blots of brain tissue (Figure 2A), with the higher band corresponding to the predicted ∼81 kDa molecular weight for the full-length protein. As both bands were similarly detected when the full-length TRIM3 cDNA was overexpressed in COS-7 cells (Figure S1A), the lower band is more likely to be a cleavage product of TRIM3 rather than an alternative spliced form or related protein.

Subcellular fractionation of rat forebrain showed TRIM3 to be particularly abundant in the light membrane fraction (P3), and sparse in the cytosol (S3) (Figure 2C). TRIM3 was also present in the Triton X-100-resistant PSD fractions (PSDI and PSD II), which corroborates its detection in the PSD by mass spectrometry [22]. However, TRIM3 is not enriched in the PSD like PSD-95 and is extracted by Sarkosyl detergent (PSDIII), indicating that TRIM3 is not tightly associated with the PSD (Figure 2B).

By immunostaining, TRIM3 was widely distributed in a micropunctate pattern throughout the cell body, axon, and dendrites of cultured hippocampal neurons (Figure 2C), consistent with its biochemical cofractionation with light membranes. This staining was diminished specifically with TRIM3 RNAi constructs and not by other shRNAs, confirming the specificity of the antibody staining (see Figure S1B). Unlike GKAP or PSD-95, TRIM3 was not concentrated in synaptic clusters. It was also undetectable in glial cells in our cultures (data not shown).

Increased neuronal activity has been reported to upregulate TRIM3 mRNA levels in the brain [27]. To examine whether TRIM3 protein levels in primary hippocampal neurons were altered by changes in activity, we treated cultures with either bicuculline (50 µM) or tetrodotoxin (TTX; 3 µM). We did not detect any significant change in TRIM3 expression with treatment times varying from 2 to 24 hours (Figure S2A). Furthermore, chronic treatment (24 h) did not affect the distribution of TRIM3 in dissociated neurons, with no change in the diffuse staining pattern (Figure S2B).

### TRIM3 is an E3 ligase for GKAP

**Figure 3:**
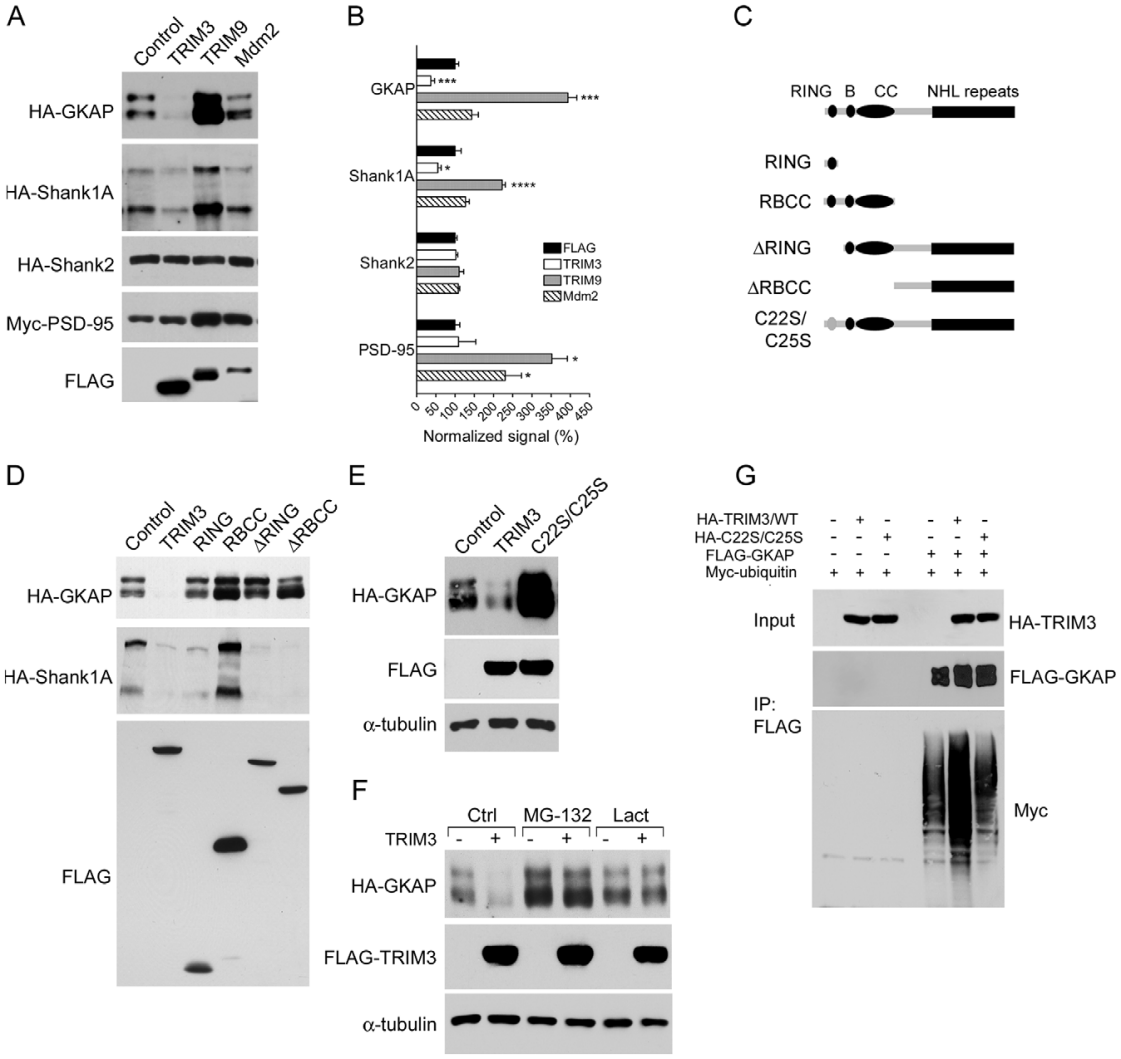
TRIM3 is a ubiquitin ligase for GKAP. (A) COS-7 cells were cotransfected with a plasmid expressing an epitope-tagged target protein (either GKAP, Shank1A, Shank2, or PSD-95) and either an empty vector (Control) or a FLAG-tagged E3 ligase (rat TRIM3, TRIM9, or human Mdm2). Cell lysates were immunoblotted for FLAG (representative example shown in bottom panel), and for the coexpressed target protein, as indicated. (B) Immunoblots were quantified by densitometry, and signals were normalized to the empty FLAG vector control. Histograms show means ± SEM for 3–6 experiments each. p-values are calculated relative to FLAG control. *p<0.05, ***p<0.001, ****p<0.0001, Student t-test. (C) Schematic diagram of TRIM3 deletion constructs, showing RING, B-box, coiled coil (CC), and NHL repeat domains. The gray oval denotes a mutated RING domain with cysteine to serine substitutions at amino acid positions 22 and 25. FLAG epitope tags are located at the amino terminus. (D) Effect of TRIM3 deletion mutants on GKAP and Shank1A expression in COS-7 cells. The lower panel shows the expression of the FLAG-tagged TRIM3 mutants, each at the expected molecular weight. (E) HA-GKAP was cotransfected with either wildtype TRIM3, or the C22S/C25S mutant TRIM3. All tested mutations abolish TRIM3-mediated GKAP degradation. Western blot of α-tubulin is shown as a loading control. (F) COS-7 cells were cotransfected with HA-GKAP and either empty FLAG vector (−) or FLAG-TRIM3 (+). Prior to harvesting, cells were left untreated or were treated either with 20 µM MG-132 or 5 µM lactacystin for 14 hours. Cell lysates were immunoblotted with either FLAG or HA antibody, with α-tubulin loading control. (G) Ubiquitination of GKAP stimulated by coexpression of wildtype, but not mutant, TRIM3. HEK293 cells were cotransfected with FLAG-GKAP, Myc-tagged ubiquitin, and either empty vector, wildtype HA-TRIM3, or the C22S/C25S mutant, as indicated. Cells were treated with MG-132 for 14 hours prior to harvesting to block proteasome-dependent GKAP degradation. Cell lysates were prepared under denaturing conditions, immunoprecipitated with FLAG antibody-coupled Sepharose, and immunoblotted as shown. As a negative control, no myc-ubiquitin signal is immunoprecipitated in the absence of FLAG-GKAP (first three lanes).

The TRIM3 RNAi phenotype implies that TRIM3 normally functions to suppress the postsynaptic levels of Shank1 protein in neurons. Assuming that TRIM3 is an E3 ligase, its effect could be mediated directly by ubiquitination/degradation of Shank1, or indirectly by degradation of another protein that influences Shank accumulation in spines. We therefore tested in heterologous cells whether overexpression of TRIM3 can stimulate the degradation of Shank1A or GKAP/SAPAP1, another abundant PSD scaffold that is implicated in Shank1 accumulation in the PSD [16], [17]. Overexpression of TRIM3 in COS7 cells reduced the protein levels of cotransfected HA-tagged Shank1A (Figure 3A, second row; quantified in B). Remarkably, TRIM3 had an even more robust effect on GKAP/SAPAP1, strongly suppressing protein expression (Figure 3A, top row). In contrast, TRIM3 had no effect on expression of PSD-95 or Shank2, a close relative of Shank1. Overexpression of TRIM9 (another neuronal TRIM protein) or Mdm2, two other single subunit E3 ligases, did not inhibit Shank1A or GKAP expression in heterologous cells. In fact, TRIM9 overexpression robustly increased protein levels of HA-GKAP, HA-Shank1A and Myc-PSD-95 (Figure 3A, B), possibly by sequestering other components required for protein degradation. Thus, in heterologous cells, TRIM3 specifically suppresses protein expression of GKAP and Shank1A, two PSD scaffolds that directly interact with each other.

In addition to the defining motifs of TRIM proteins (RING finger, B-Box, and coiled coil), TRIM3 contains a series of six NHL repeats (first identified in NCL-1, HT2A, and LIN-41) [28] (see Figure 3C). These repeats are similar to WD repeats, which are known to be involved in protein-protein interaction and are the substrate-recruiting module of many E3 ligases [29]. To determine which regions of TRIM3 are required for the suppression of Shank1A and GKAP, we examined the effects of several deletion mutants lacking various domains of TRIM3 (Figure 3C). All of these mutant constructs lost the ability to reduce GKAP expression in COS7 cells (Figure 3D). From this experiment, we conclude that the RING finger domain and the C-terminal half of TRIM3 containing the NHL repeats are both required for TRIM3 to suppress GKAP protein levels. Because the RING finger domain plays a critical role in E3 ligase function by mediating the transfer of ubiquitin to target substrates [30], our data are consistent with TRIM3 acting as an E3 ubiquitinating enzyme for GKAP.

RING finger structure and function requires zinc coordination by conserved cysteine and/or histidine residues [30]. Mutation of the first two conserved cysteine residues (C22S/C25S) abolished the ability of TRIM3 to suppress GKAP protein levels (Figure 3E). In fact, GKAP expression was increased by coexpression of C22S/C25S-TRIM3 compared to the negative control (empty vector), suggesting a dominant negative effect of this mutant. In addition, the loss of GKAP with TRIM3 cotransfection was blocked by addition of the proteasome inhibitors MG-132 or lactacystin (Figure 3F). These data strongly support the idea that TRIM3 suppresses GKAP protein expression by stimulating UPS-mediated degradation of GKAP.

Surprisingly, the TRIM3 deletion constructs ΔRING, ΔRBCC, and RING still resulted in loss of Shank1A protein in COS-7 cells, even though these mutants should be defective as E3 ligases and were incapable of suppressing GKAP protein expression (Figure 3D). In addition, proteasomal inhibition by MG-132 did not fully prevent the decrease in Shank1A induced by cotransfection of TRIM3 (data not shown). At least in heterologous cells, therefore, the inhibition of Shank1A protein levels by TRIM3 appears to be unrelated to the E3 ligase activity of TRIM3. Overall, our data indicate that GKAP is more likely than Shank1A to be a conventional substrate of TRIM3-mediated UPS degradation.

If TRIM3 is a genuine E3 for GKAP, it should be able to stimulate ubiquitination of GKAP. We cotransfected COS7 cells with FLAG-GKAP, Myc-tagged ubiquitin, and either wildtype TRIM3 or the C22S/C25S mutant (Figure 3G). Cells were treated with MG-132 to prevent substrate degradation, and GKAP was immunoprecipitated with FLAG antibodies and immunoblotted for Myc-ubiquitin. In cells expressing wildtype TRIM3, there was a marked increase in the amount of myc-ubiquitinated GKAP, compared to cells lacking TRIM3 or expressing the C22S/C25S mutant. In the absence of FLAG-GKAP, no Myc signal was immunoprecipitated by the FLAG antibody, indicating that the Myc-ubiquitin is specifically associated with GKAP, rather than non-specifically immunoprecipitated. Collectively, these data suggest that TRIM3 stimulates GKAP degradation by acting as a specific ubiquitin ligase for GKAP.

### TRIM3 regulates GKAP and Shank1 expression in hippocampal neurons

**Figure 4:**
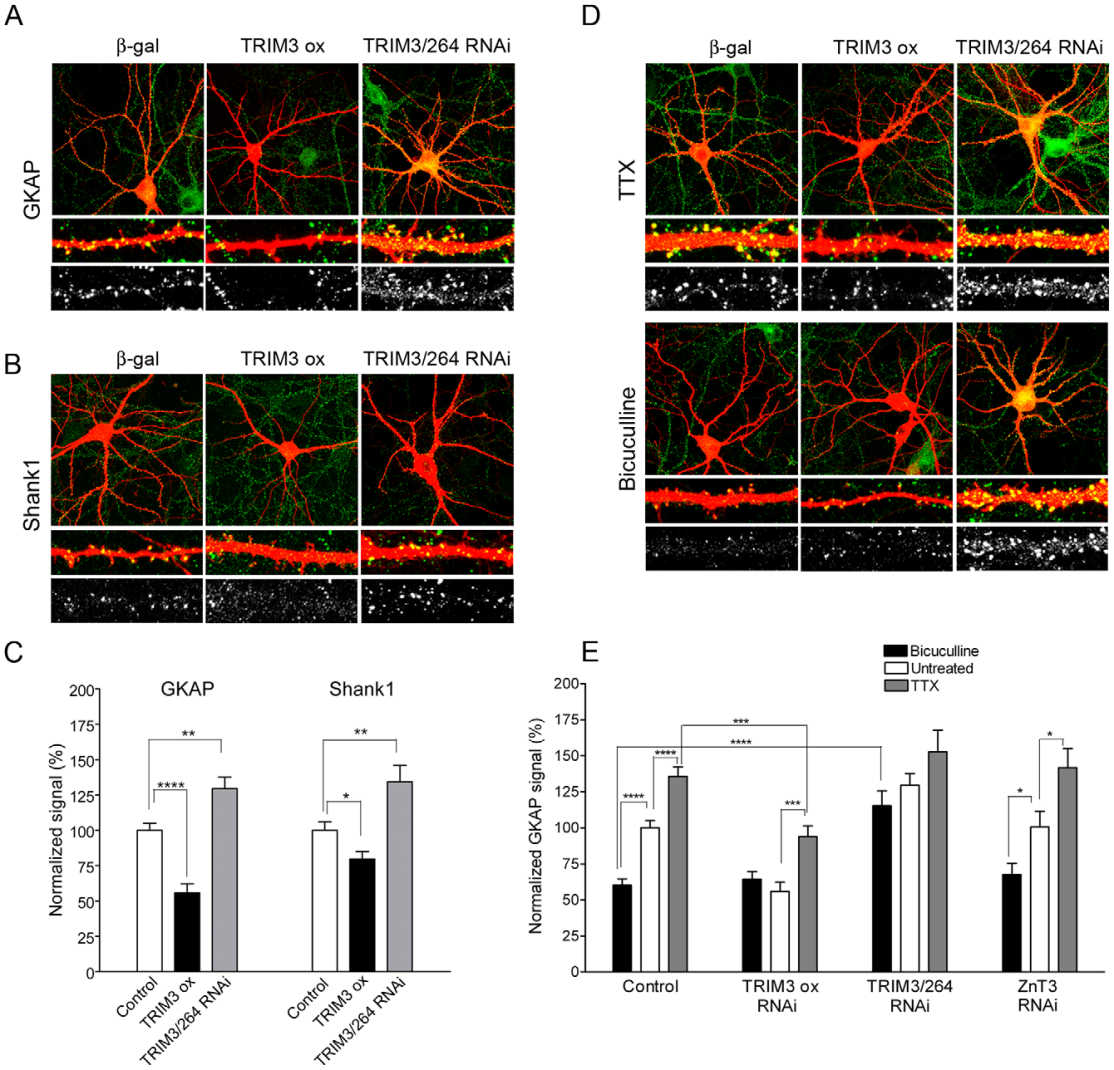
TRIM3 modulates basal and activity-regulated GKAP levels in hippocampal neurons. (A) Rat hippocampal neurons were transfected at DIV14 with β-galactosidase (β-gal) and either empty vector, wildtype TRIM3, or TRIM3/264 RNAi plasmid. After four days, cells were double-labeled for GKAP (green) and either β-gal or the overexpressed TRIM3 protein (red). Representative neurons are shown, with higher magnification images of dendritic segments. The bottom row shows the GKAP (green) channel in grayscale. (B) Similar experiment to (A), with cells double-labeled for Shank1 (green) and either β-gal or the overexpressed TRIM3 protein (red). (C) Quantification of endogenous GKAP or Shank1 immunofluorescence upon TRIM3 overexpression (TRIM3 ox) or knockdown with TRIM3/264 RNAi. Intensity is normalized to cells transfected with empty vector. (D) Neurons were transfected at DIV14 with β-galactosidase (β-gal) and either empty vector, wildtype TRIM3, TRIM3 RNAi plasmid, or an unrelated RNAi (ZnT3). After three days, cells were treated for an additional 24 hours with either TTX (3 µM) or bicuculline (50 µM) prior to staining. (E) Quantification of endogenous GKAP immunofluorescence intensity upon TRIM3 overexpression or knockdown, in untreated neurons or after TTX or bicuculline treatment. The data used for untreated cells is the same as that shown in (C), replotted here for comparison with TTX and bicuculline treated conditions. Histograms show mean ± SEM for a minimum of 13 neurons per condition. *p<0.05, **p<0.01, ***p<0.001; ****p<0.0001, t-test.

We next examined whether TRIM3 functions in neurons to regulate expression of endogenous GKAP protein. Cultured hippocampal neurons were cotransfected at DIV 14 with a β-galactosidase marker and TRIM3 constructs, and immunostained four days later for endogenous GKAP. In neurons overexpressing TRIM3, levels of dendritic GKAP were significantly reduced (Figure 4A, quantified in C). Conversely, RNAi knockdown of TRIM3 (TRIM3/264 RNAi) resulted in increased GKAP immunoreactivity. The intensity of endogenous Shank1 immunostaining also showed bidirectional changes with TRIM3 overexpression and TRIM3 RNAi, similar to GKAP (Figure 4B, C). The increase of endogenous Shank1 with TRIM3 knockdown is in keeping with the effect on exogenous GFP-Shank1A in the original RNAi screen (see Figure 1). These gain- and loss-of-function data show that TRIM3 modulates the postsynaptic levels of GKAP and Shank1 scaffolds endogenously in neurons.

The abundance of GKAP in the PSD is regulated bidirectionally over many hours by neuronal activity [3]. In cultured hippocampal neurons transfected with β-gal alone, suppressing activity with tetrodotoxin (TTX, 3 µM, 24 h) resulted in higher levels of GKAP staining in dendrites, whereas stimulating activity with bicuculline (50 µM, 24 h) reduced GKAP protein expression (Figure 4D, quantified in E). Similar results were obtained in neurons transfected with a control RNAi construct targeting ZnT3, a zinc transporter. In cells overexpressing wildtype TRIM3, endogenous GKAP staining was depleted and treatment with bicuculline had no additional lowering effect. Thus, TRIM3 overexpression “occluded” the effect of hyperactivity on GKAP levels. Although TRIM3 overexpression by itself inhibited GKAP protein expression, it did not prevent an increase of GKAP expression induced by TTX. This latter result suggests that TRIM3 function may be itself under activity-dependent control, or there are alternate ways to increase GKAP expression that are independent of TRIM3.

In neurons expressing TRIM3/264 shRNA, bicuculline treatment failed to lower GKAP protein expression, despite a higher “baseline” level of GKAP (Figure 4D, E). Conversely, TRIM3 RNAi had no additional effect on elevation of GKAP expression by TTX. The simplest interpretation of these results is that TRIM3 is required for activity-induced downregulation of GKAP, and loss of TRIM3 occludes the upregulation of GKAP by chronic hypoactivity (TTX). Overall, we conclude that endogenous TRIM3 is important for activity-dependent regulation of postsynaptic GKAP levels.

### TRIM3 modulates dendritic spine morphology

**Figure 5:**
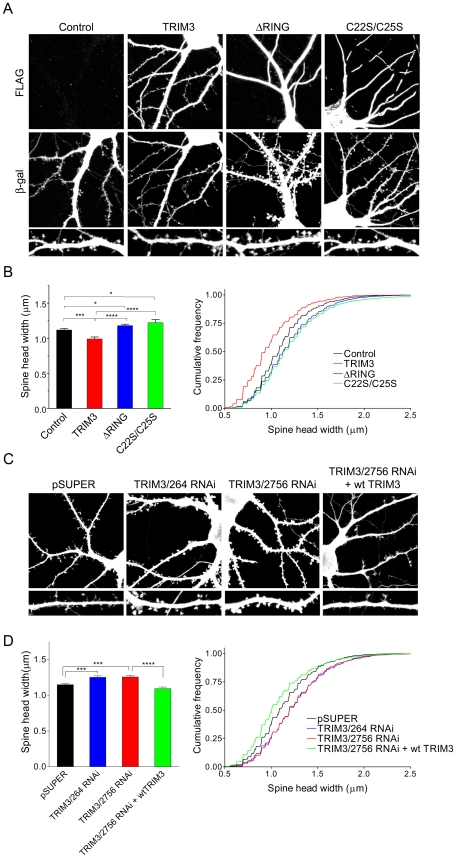
Regulation of dendritic spine morphology by TRIM3. (A) Cultured hippocampal neurons were cotransfected at DIV14 for three days with either empty FLAG vector (Control), FLAG-TRIM3 (wildtype), or TRIM3 mutants defective in RING finger function (ΔRING or C22S/C25S) along with β-galactosidase (β-gal) to visualize cell morphology. Cells were double-labeled for the FLAG-tagged protein and β-gal. Representative transfected neurons are shown, with higher magnification images of dendritic segments (labeled for β-gal). (B) Quantification of spine head width upon overexpression of wildtype or mutant TRIM3. Cumulative frequency distribution is shown at right. (C) Effect of TRIM3 knockdown by RNAi on dendritic spines. DIV14 hippocampal neurons cotransfected with β-gal and either pSUPER, TRIM3/264 or TRIM3/2756 RNAi constructs are shown, with representative dendritic segments. The effect of TRIM3/2756 is negated by the overexpression of wildtype TRIM3. (D) Quantification of spine head width from cells transfected as in (C), with cumulative frequency distribution shown at right. Spines from at least 13–14 cells (overexpression) or 24 cells (RNAi) were quantified. *p<0.05, ***p<0.001, ****p<0.0001, t-test. Each cumulative frequency distribution of spine head width (B and D) is significantly different from control (p<0.0001, Kolmogorov-Smirnov test).

PSD scaffold proteins are known to regulate dendritic spine morphology [6], [25], [31]. We therefore asked whether TRIM3, as an E3 ligase, has any role in regulating dendritic spine morphogenesis. Dissociated hippocampal neurons (DIV14) were transfected for 3 days with β-galactosidase (to visualize spine morphology) and either wildtype TRIM3 or mutants ΔRING or C22S/C25S. Compared with vector-transfected cells, neurons overexpressing wildtype TRIM3 showed a decrease in spine head width, while those overexpressing the putative dominant negative TRIM3 mutants had enlarged spine heads (Figure 5A, B) There was little effect on spine length (Figure S3A) or spine density (spines/10 µm ± SEM; Control: 4.79±0.18; TRIM3 4.88±0.22; ΔRING 5.36±0.29; C22S/C25S 4.87±0.21). We noted that deletion or mutation of the RING finger domain altered the distribution of the overexpressed protein (Figure 5A). Whereas the overexpressed wildtype protein was present diffusely within the cell including dendritic spines, the mutant TRIM3 proteins showed a “fibrillar” pattern in the dendrite shaft and were excluded from spines.

To corroborate the dominant negative experiments, we analyzed the effects on spine morphology of two different TRIM3 RNAi constructs (TRIM3/264, directed against part of the coding sequence, and TRIM3/2756, targeted against the 3′ untranslated region). Similar to the RING finger mutants, both TRIM3 RNAi plasmids induced a significant increase in dendritic spine head width (Figure 5C, D). When FLAG-tagged TRIM3 was coexpressed with TRIM3/2756 shRNA (FLAG-TRIM3 plasmid does not contain the 3′ UTR, and is therefore insensitive to knockdown by TRIM3/2756 RNAi), the phenotype shifted back toward thinner spines. This “rescue” experiment suggests that the enlargement of spine heads is a specific consequence of TRIM3 knockdown, and not an off-target effect of the shRNA. RNAi knockdown of TRIM3 did not greatly influence spine length (Figure S3B) or density (spines/10 µm; pSUPER: 5.34±0.18; TRIM3/264 6.18±0.24; TRIM3/2756 5.02±0.29; TRIM3/2756  =  wt TRIM3 4.52±0.18). Taken together, our findings imply that TRIM3 functions normally to inhibit growth of dendritic spine heads, dependent on its ubiquitin ligase activity.

## Discussion

Many proteins of the PSD are dynamically regulated by translocation to and from the synapse and by degradation [1], [2]. A subset of these PSD proteins show coordinated bidirectional changes in abundance in response to chronically altered activity mediated by the UPS [3]. However, the E3 ligases that govern activity-dependent turnover of specific PSD components have not been identified. In this study, we identify TRIM3 as an E3 ligase that selectively targets GKAP, based on the following evidence. (i) TRIM3 stimulates the ubiquitination and loss of GKAP in heterologous cells, dependent on the RING finger of TRIM3 and on proteasome function; (ii) overexpression of TRIM3 leads to the depletion of endogenous GKAP in neurons; (iii) RNAi knockdown of endogenous TRIM3 in neurons results in elevated levels of postsynaptic GKAP. The presence of TRIM3 in PSD fractions (initially discovered by unbiased proteomic approaches [22]) lends further support to the idea that TRIM3 has a role at postsynaptic sites.

RNAi knockdown of TRIM3 also resulted in increased abundance of Shank at postsynaptic sites, which is in keeping with the original RNAi screen that identified TRIM3 and which used GFP-Shank1 as the “reporter”. This fact notwithstanding, our data are more consistent with the idea that GKAP is the direct target of TRIM3 and that the effect of TRIM3 on Shank1 expression occurs secondarily to GKAP loss. Although TRIM3 suppressed protein levels of Shank1 in heterologous cells, this effect was surprisingly not dependent on the presence of the RING finger domain and was not completely blocked by proteasome inhibitors, in contrast to suppression of GKAP by TRIM3. Thus, these data are not consistent with Shank1 as a direct substrate for TRIM3. It is known that Shank proteins bind directly to GKAP through an interaction between the Shank PDZ domain and the C-terminus of GKAP. In neurons, disrupting this interaction results in the loss of Shank from synapses and accumulation of Shank within the cell body [16], [17]. Thus, the parallel changes in Shank1 upon manipulation of TRIM3 could be explained by decreased stabilization of Shank at the PSD secondary to GKAP loss.

Based on its interaction with myosin Vb, TRIM3 has been proposed to be involved in organelle or vesicle transport [13], [32]. It is intriguing that GKAP itself can associate with myosin V via its dynein light chain subunit [33]. Myosin V could thus bring together TRIM3 and its substrate GKAP, or mediate the cotrafficking of these proteins. Our biochemical data suggest that TRIM3 plays a direct role in UPS-mediated GKAP loss, but we cannot exclude that TRIM3 also regulates the trafficking of GKAP to and from synapses.

In neurons, chronic enhancement of synaptic activity with bicuculline treatment increases the level of ubiquitinated GKAP [3] and leads to depletion of synaptic GKAP in a TRIM3-dependent manner (this study). The increased ubiquitination and turnover of GKAP by activity implies that the target protein (GKAP) and/or the UPS system must be modified by neuronal excitation. Elevated activity could potentially induce phosphorylation of GKAP, marking it for destruction. Phosphorylation-dependent recruitment of E3 ligases to substrates is a well-established means of regulating ubiquitination of specific substrates [34]. It is noteworthy that TRIM3 contains C-terminal NHL repeats, a protein motif that resembles the WD repeats found in many F-box proteins, the substrate recognition component of multisubunit SCF (Skp-1/cullin/F-box) E3 ligases. WD repeats can specifically recognize phosphorylated substrates for UPS-mediated degradation [35]–[37]. Synaptic activity stimulates a number of protein kinases, which could potentially mark GKAP for degradation. The identity of such a kinase and the site of phosphorylation remain to be investigated.

Alternatively, activity could affect the expression, function or subcellular localization of TRIM3, thereby stimulating GKAP ubiquitination-degradation. Indeed, increased activity stimulates TRIM3 mRNA expression in the brain [27]. However, we observed no major changes in TRIM3 protein expression or localization in dissociated hippocampal cultures in response to chronic TTX or bicuculline. In addition, neuronal activity promotes translocation of proteasomes into dendritic spines, thereby delivering the protein degradation machinery to its intended synaptic targets [38]. One of our findings suggesting activity-dependent regulation of TRIM3 function is that TRIM3 RNAi increases dendritic GKAP levels under conditions of basal (control) or increased synaptic activity (bicuculline), but has no significant effect when neurons are silenced by TTX (see Figure 4). One possible explanation is that TRIM3 enzymatic function is stimulated by activity and inactivated in the presence of chronic TTX.

We also show here that TRIM3 negatively regulates the size of spine heads, with loss of function resulting in increased spine size. The morphological changes are presumably a postsynaptic effect, as they are observed in the spines of transfected cells in which TRIM3 expression has been altered. The mechanism likely involves the direct or indirect loss of PSD scaffold proteins such as GKAP and Shank, as Shank1 strongly promotes spine head enlargement [25]. Regulation of excitatory synapses may depend on a complex balance between opposing sets of E3 ligases, as different E3s have been reported to either increase or decrease synaptic size [39], [40]. TRIM3 is likely to have additional postsynaptic substrates, and based on its presence in axons, may also have a presynaptic role.

Recent studies have demonstrated that dendritic spine dimensions are bidirectionally regulated by synaptic activity, and that synaptic transmission and calcium signaling is determined by the shape of individual spines [41]. Thus, E3 ligases such as TRIM3 that regulate postsynaptic GKAP and Shank content could play important roles in synaptic function and plasticity. Interestingly, a recent study demonstrates that GKAP and Shank are polyubiquitinated after retrieval of contextual fear memory [42]. Furthermore, mutations in members of the GKAP/SAPAP and Shank family of genes may contribute to obsessive-compulsive and autism spectrum disorders, respectively [43]–[45], indicating that proper regulation of the turnover of these proteins is critical for normal cognitive functions.

## Methods

### RNA interference screen

Two to three annealed sets of oligonucleotides encoding small hairpin RNAs targeting unique sequences for each E3 ligase component (see Table S1) were cloned individually into pSUPER [23]. The GFP-Shank1A reporter construct contained the entire rat Shank1A coding region and 523 bp of the 3′ untranslated region shown to mediate dendritic targeting of the transcript [46], under the control of the ubiquitin promoter. The GFP-Shank1A plasmid was cotransfected into primary rat hippocampal neurons (DIV 14) with pools of RNAi plasmids at a DNA mass ratio of 1∶3. After three days, cultures were fixed with 4% paraformaldehyde/4% sucrose, and GFP fluorescence was imaged directly by confocal microscopy. GFP-Shank1A signal was measured by integrating GFP fluorescence intensity and normalizing to cells transfected with pSUPER control. The mean number of cells analyzed per target (n) was 44 (range 25–69), taken from 3–4 independent experiments. The PSD-95-GFP construct has been described previously [47].

### DNA constructs

The following mammalian expression plasmids have been described: pGW1/HA-GKAP [48], pGW1/HA-Shank1A [25], pGW1/Myc-PSD-95 [49]. COS-7 cell experiments were performed using a construct encoding the full-length short form of GKAP/SAPAP1 [48]. Full-length rat TRIM3, TRIM2, and TRIM9 cDNAs were isolated by PCR from a rat brain cDNA library, cloned into a modified pGW1 vector (British Biotechnology) containing an N-terminal FLAG epitope, and confirmed by direct sequencing. Deletion mutants of TRIM3 were generated by PCR, and the C22S/C25S mutant was generated by site-directed mutagenesis using the QuikChange method (Stratagene). To generate doxycycline-inducible TRIM3 and TRIM2 expression constructs, full-length cDNAs were cloned into a modified pTRE vector (Clontech).

### Antibodies and biochemistry

Rabbit GKAP (C9589) and guinea pig PSD-95 antibodies have been described previously [48], [50]. The GKAP antibody was generated against the conserved C-terminus and recognizes all isoforms. Mouse PSD-95 (clone K28/43) was a gift from J. Trimmer (UC-Davis). The following antibodies were obtained from commercial sources: mouse anti-BERP/TRIM3 (BD Biosciences); rabbit anti-Shank1A (Chemicon); rabbit (ICN), mouse (Promega) anti-β-galactosidase; rabbit anti-HA, mouse anti-myc (Santa Cruz); rabbit and mouse anti-FLAG (Sigma); Alexa488-, Alexa568-conjugated secondary antibodies (Molecular Probes). Tetrodotoxin, bicuculline, and MG-132 were obtained from Sigma.

Biochemical fractionation of rat forebrain was performed as described previously [51]. COS-7 cells were transfected using Lipofectamine (Invitrogen). For immunoprecipitation, cells were extracted with buffer A (50 mM Tris, ph 7.4, 150 mM NaCl, 5 mM EDTA, 5 mM EGTA, protease inhibitors) containing 2% SDS at 4°C. for 1 hr. After centrifugation at 16,000 g for 30 minutes, extracts were diluted with 9 volumes of buffer A containing 1% Triton, and then immunoprecipitated with FLAG M2-agarose (Sigma).

### Primary hippocampal neuron culture, transfection and immunocytochemistry

Primary hippocampal neuron cultures were prepared from embryonic day 19 (E19) rat hippocampi, and plated onto glass coverslips coated with poly-D-lysine (30 µg/ml) and laminin (2 µg/ml) at a concentration of 150–200 cells/mm^2^, as described previously [52]. Cultured neurons were transfected on DIV14 using Lipofectamine 2000 (Invitrogen). For spine experiments, β-galactosidase and RNAi plasmids were cotransfected at a ratio of 1∶3. In rescue experiments with wildtype TRIM3, ratio of β-galactosidase:FLAG-TRIM3:RNAi plasmids was 1∶1∶3. Tetrodotoxin (3 µM) or bicuculline (50 µM) was added three days after transfection, and cells were incubated for an additional 24 hr.

For immunocytochemistry, neurons were fixed with 4% paraformaldehyde (PFA)/4% sucrose for analysis of cell morphology, or briefly with 4% PFA followed by methanol (−20°C) for immunofluorescent labeling of synaptic proteins. Primary and secondary antibodies were applied in GDB buffer (30 mM phosphate buffer, pH 7.4, containing 0.2% gelatin, 0.45 M NaCl, 0.5% Triton X-100).

### Image analysis and quantification

Confocal images of transfected neurons were obtained with a LSM510 confocal microscope (Zeiss) using a Zeiss 63x (N.A. 1.4) oil objective with sequential acquisition settings of 1024×1024 pixels. Each image was a z-series projection of 6–10 images, each averaged 2 times and taken at 0.36 µm depth intervals. When fluorescence intensity was compared, confocal settings were kept unchanged within a given experiment. Fluorescence quantification and morphometric analysis were performed using MetaMorph software (Universal Imaging). Fluorescence intensity of endogenous GKAP and Shank1 was determined from dendritic segments totaling at least 100–150 µm per cell. Randomly selected dendritic segments were carefully traced, and the integrated intensity and area were determined for each segment. The average intensity was then determined for each cell, and normalized to control transfected cells (β-gal plus either empty FLAG vector or pSUPER). Spine measurements were determined from 3–5 dendritic segments per cell, as previously described [25]. Acquisition and quantification of images were performed under “blinded” conditions. Statistical analysis was performed with t-test, defining n as the number of transfected neurons per condition.

## Supporting Information

Figure S1 Specific knockdown of TRIM3 by RNA interference. (A) Specificity of TRIM3 antibody. COS-7 cells were transfected with plasmids expressing either TRIM3, TRIM2 or TRIM9, each tagged at the amino terminus with the FLAG epitope. Cell lysates were blotted with anti- FLAG and TRIM3 antibodies. (B) Hippocampal neurons were cotransfected with beta-galactosidase and either pSUPER or RNAi constructs targeting TRIM3, TRIM2, the unrelated protein ZnT3 at DIV14. After three days, cells were briefly fixed with paraformaldehyde followed by methanol, and then immunostained for beta-galactosidase (red) and endogenous TRIM3 (green). As an additional control, a neuron cotransfected with wildtype TRIM3 and the TRIM3/2756 RNAi construct is shown, rescuing knockdown of the endogenous protein. Under these conditions, beta-galactosidase staining does not accurately reflect cell morphology. (C) COS-7 cells were triply transfected with RNAi plasmids targeting either TRIM3 or the related gene TRIM2, cDNAs expressing TRIM3 (upper panel) or TRIM2 (lower panel) under the control of a doxycycline-inducible promoter, and a plasmid encoding a doxycycline-dependent transcriptional activator. After treatment with doxycycline to induce the TRIM transcription, cells were lysed and immunoblotted with FLAG antibody to detect the transfected TRIM protein.
(1.88 MB TIF)

Figure S2 Expression of TRIM3 in cultured hippocampal neurons in response to changes in activity. (A) DIV17 hippocampal neurons were treated with either bicuculline (50 micromolar) or TTX (3 micromolar) for the indicated times. Total cell lysates were immunoblotted for TRIM3. (B) Hippocampal neurons were treated with either bicuculline or TTX for 24 hr. There is no change in TRIM3 expression or localization. In contrast, GKAP and PSD-95 expression is downregulated by bicuculline and upregulated by TTX.
(0.87 MB TIF)

Figure S3 Effect of TRIM3 perturbation on dendritic spine length. (A) Quantification of dendritic spine lengths from cultured hippocampal neurons cotransfected with beta-galactosidase and either empty FLAG vector (Control), FLAG-TRIM3 (wildtype), or TRIM3 mutants defective in RING finger function (Delta-RING or C22S/C25S) (representative cells shown in Figure 5A). (B) Effect of TRIM3 knockdown by RNAi on dendritic spine length (representative cells shown in Figure 5C). Bar graphs show mean +/− SEM. Cumulative frequency distributions are shown at right.
(0.38 MB TIF)

Table S1 RNAi targeting sequences used in this study.
(0.05 MB DOC)
