# Supplementary figures and images for: Degradation of Postsynaptic Scaffold GKAP and Regulation of Dendritic Spine Morphology by the TRIM3 Ubiquitin Ligase in Rat Hippocampal Neurons
Source: PLoS One. 2010 Mar 24;5(3):e9842. doi: 10.1371/journal.pone.0009842 (PMC2844417; doi:10.1371/journal.pone.0009842)

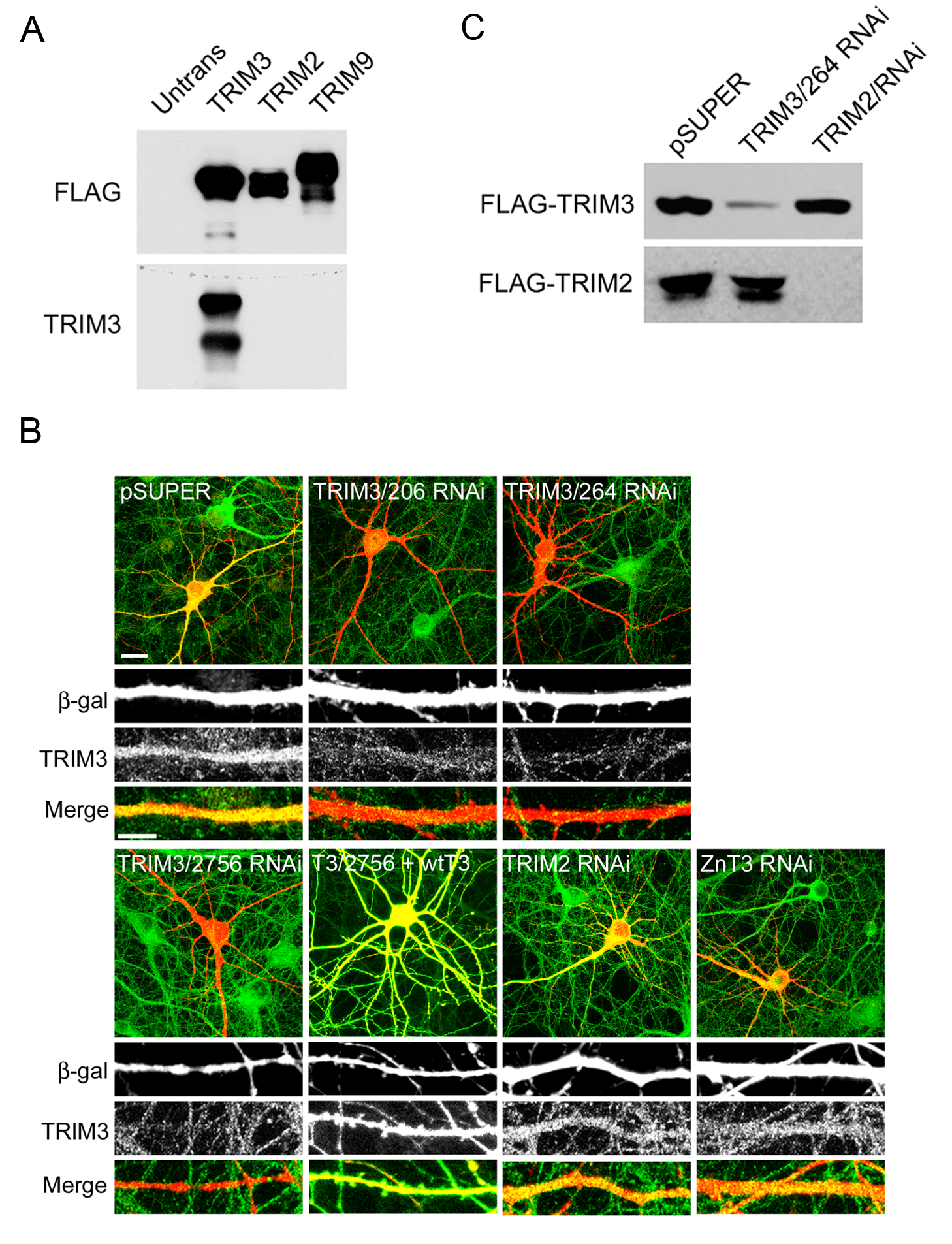

Supplement: Figure S1 — Specific knockdown of TRIM3 by RNA interference. (A) Specificity of TRIM3 antibody. COS-7 cells were transfected with plasmids expressing either TRIM3, TRIM2 or TRIM9, each tagged at the amino terminus with the FLAG epitope. Cell lysates were blotted with anti- FLAG and TRIM3 antibodies. (B) Hippocampal neurons were cotransfected with beta-galactosidase and either pSUPER or RNAi constructs targeting TRIM3, TRIM2, the unrelated protein ZnT3 at DIV14. After three days, cells were briefly fixed with paraformaldehyde followed by methanol, and then immunostained for beta-galactosidase (red) and endogenous TRIM3 (green). As an additional control, a neuron cotransfected with wildtype TRIM3 and the TRIM3/2756 RNAi construct is shown, rescuing knockdown of the endogenous protein. Under these conditions, beta-galactosidase staining does not accurately reflect cell morphology. (C) COS-7 cells were triply transfected with RNAi plasmids targeting either TRIM3 or the related gene TRIM2, cDNAs expressing TRIM3 (upper panel) or TRIM2 (lower panel) under the control of a doxycycline-inducible promoter, and a plasmid encoding a doxycycline-dependent transcriptional activator. After treatment with doxycycline to induce the TRIM transcription, cells were lysed and immunoblotted with FLAG antibody to detect the transfected TRIM protein. (1.88 MB TIF) [file pone.0009842.s001.tif]

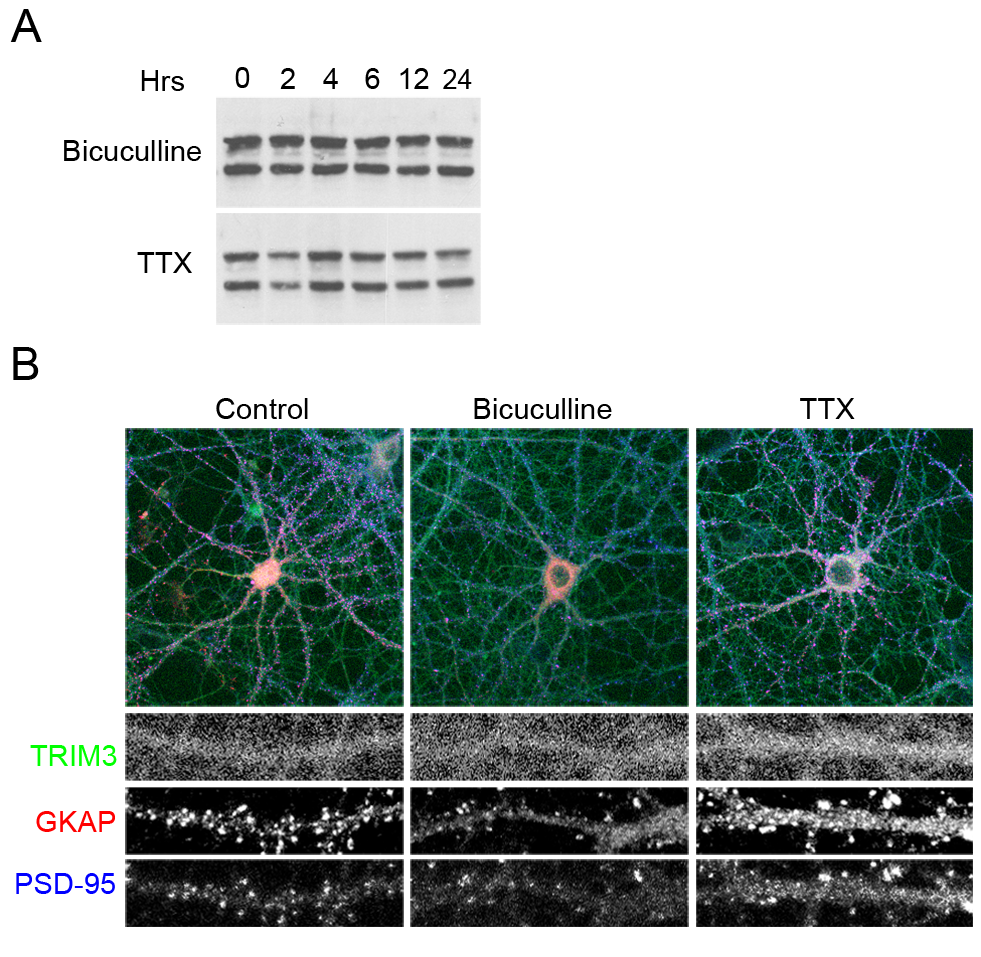

Supplement: Figure S2 — Expression of TRIM3 in cultured hippocampal neurons in response to changes in activity. (A) DIV17 hippocampal neurons were treated with either bicuculline (50 micromolar) or TTX (3 micromolar) for the indicated times. Total cell lysates were immunoblotted for TRIM3. (B) Hippocampal neurons were treated with either bicuculline or TTX for 24 hr. There is no change in TRIM3 expression or localization. In contrast, GKAP and PSD-95 expression is downregulated by bicuculline and upregulated by TTX. (0.87 MB TIF) [file pone.0009842.s002.tif]

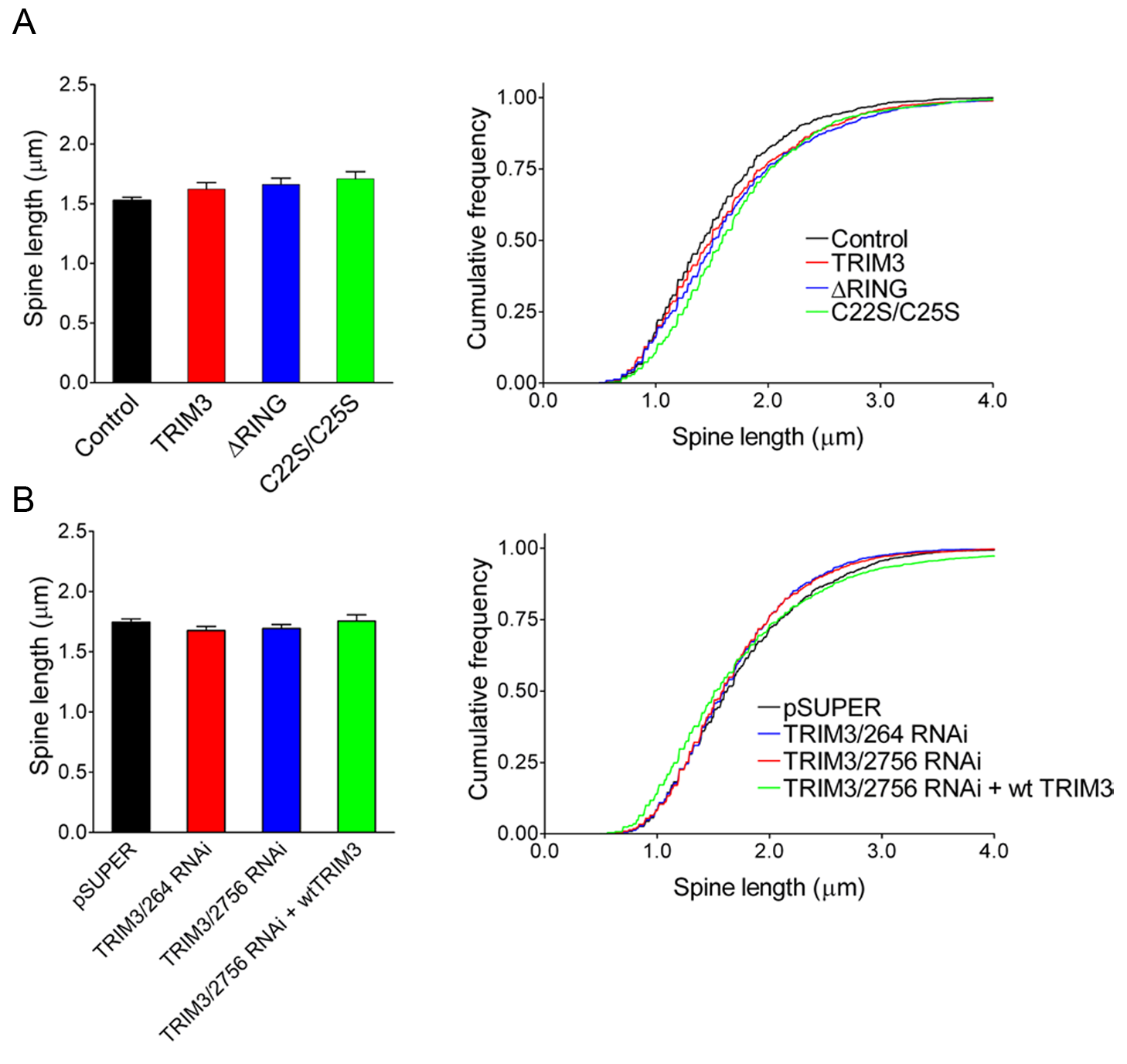

Supplement: Figure S3 — Effect of TRIM3 perturbation on dendritic spine length. (A) Quantification of dendritic spine lengths from cultured hippocampal neurons cotransfected with beta-galactosidase and either empty FLAG vector (Control), FLAG-TRIM3 (wildtype), or TRIM3 mutants defective in RING finger function (Delta-RING or C22S/C25S) (representative cells shown in Figure 5A). (B) Effect of TRIM3 knockdown by RNAi on dendritic spine length (representative cells shown in Figure 5C). Bar graphs show mean +/− SEM. Cumulative frequency distributions are shown at right. (0.38 MB TIF) [file pone.0009842.s003.tif]
