# Supplementary material for: Degradation of Postsynaptic Scaffold GKAP and Regulation of Dendritic Spine Morphology by the TRIM3 Ubiquitin Ligase in Rat Hippocampal Neurons
Source: PLoS One. 2010 Mar 24;5(3):e9842. doi: 10.1371/journal.pone.0009842 (PMC2844417; doi:10.1371/journal.pone.0009842)
Supplement: Table S1 — RNAi targeting sequences used in this study. (0.05 MB DOC) [file pone.0009842.s004.doc]

Table 1. RNAi Targeting Sequences Used in This Study

| **RNAi** | **GenBank accession number/Reference** | **Targeted Sequence** |
| --- | --- | --- |
| Cul1/404 | NM_001108627 | CATTCTTTCTGAGCTGCTG |
| Cul1/526 | NM_001108627 | TGTCATCAAACAGGAGTCA |
| Cul1/3070 | NM_001108627 | CTCACAGACAGAGGGGAAC |
| Cul2/348 | NM_001108417 | CCACTTGGAGAAAGACTTT |
| Cul2/416 | NM_001108417 | GAGAGTTCTGGAGTCTGAA |
| Cul2/2245 | NM_001108417 | TCAGCCAGTCAAGAGCCAG |
| Cul3/132 | XM_217454 | TCTGAGCAAAGGCACGGGC |
| Cul3/159 | XM_217454 | GGACACCAAGATGCGGATC |
| Cul3/2393 | XM_217454 | CACCTGAGGATCGCAAAGT |
| Cul4A/254 | XM_341464 | GCTGGTCATCAAGAACTTC |
| Cul4A/299 | XM_341464 | CTACACTCAGGACACGTGG |
| Cul4A/3253 | XM_341464 | CGAGGCTCCATAGTGGTGT |
| Cul5/135 | NM_022683 | GTTGAGAACATGGCGACGT |
| Cul5/192 | NM_022683 | GACAAGTGGGATTTCATGC |
| Cul5/2654 | NM_022683 | TCCCTTCATGTTGCACACT |
| TRIM3/206 | NM_031786 | CCATCTGTGGTCACCATGG |
| TRIM3/264 | NM_031786 | TGGACAAGCAGTTTCTGGT |
| TRIM3/2756 | NM_031786 | CAGCCTCTTACTTCAGAAG |
| TRIM9/69 | NM_130420 | GGTGCTGGACACCACAGGC |
| TRIM9/186 | NM_130420 | TGCCCCGTGTGTGGCTCCT |
| TRIM9/2068 | NM_130420 | CTGAAGGAGGGATCACAAA |
| APC2/167 | XM_215994 | TACTGTGAGCACCGGTCTG |
| APC2/2495 | XM_215994 | GAAGGTCCGAGACCAGCAG |
| TRIM2 | NM_001108552 | CAGTTCATCTCAGAAATCA |
| ZnT3 | Reference 52 | GGGCATGGATACCCAATGT |
